# Endoglin Modulates TGFβR2 Induced VEGF and Proinflammatory Cytokine Axis Mediated Angiogenesis in Prolonged DEHP-Exposed Breast Cancer Cells

**DOI:** 10.3390/biomedicines10020417

**Published:** 2022-02-10

**Authors:** Mahendra Jadhao, Chun-Lin Chen, Wangta Liu, Dhanashri Deshmukh, Wei-Ting Liao, Jeff Yi-Fu Chen, Ritesh Urade, Eing-Mei Tsai, Sheng-Kai Hsu, Li-Fang Wang, Chien-Chih Chiu

**Affiliations:** 1 Department of Medicinal and Applied Chemistry, Kaohsiung Medical University, Kaohsiung 807, Taiwan; mah.jadhao@yahoo.com (M.J.); dhanashrivdeshmukh1990@gmail.com (D.D.); 2 Department of Biological Sciences, National Sun Yat-Sen University, Kaohsiung 804, Taiwan; chunlinchen@mail.nsysu.edu.tw (C.-L.C.); uraderit@gmail.com (R.U.); 3 Graduate Institute of Natural Products, College of Pharmacy, Kaohsiung Medical University, Kaohsiung 807, Taiwan; 4 Department of Biotechnology, Kaohsiung Medical University, Kaohsiung 807, Taiwan; liuwangta@kmu.edu.tw (W.L.); wtliao@kmu.edu.tw (W.-T.L.); yifuc@kmu.edu.tw (J.Y.-F.C.); b043100050@gmail.com (S.-K.H.); 5 Department of Obstetrics and Gynecology, Kaohsiung Medical University Hospital, Kaohsiung 807, Taiwan; tsaieing@kmu.edu.tw; 6 The Graduate Institute of Medicine, College of Medicine, Kaohsiung Medical University, Kaohsiung 807, Taiwan; 7 Department of Medical Research, Kaohsiung Medical University Hospital, Kaohsiung 807, Taiwan; 8 Center for Cancer Research, Kaohsiung Medical University Hospital, Kaohsiung Medical University, Kaohsiung 807, Taiwan

**Keywords:** DEHP, angiogenesis, endoglin, VEGF, TGFβ signaling, inflammatory cytokines

## Abstract

Angiogenesis is the process of vascular network development and plays a crucial role in cancer growth, progression, and metastasis. Phthalates are a class of environmental pollutants that have detrimental effects on human health and are reported to increase cancer risk. However, the interplay between phthalate exposure and angiogenesis has not been investigated thoroughly. In this study, we investigated the effect of prolonged di (2-ethylhexyl) phthalate (DEHP) treatment on the angiogenic potential of triple-negative breast cancer. MDA-MB-231 cells were exposed to physiological concentrations of DEHP for more than three months. Prolonged DEHP exposure induced angiogenesis in breast cancer cells. Endoglin (ENG)/CD105 is a membrane glycoprotein and an auxiliary receptor of the TGFβ receptor complex. In endothelial cells, ENG is highly expressed and it is a prerequisite for developmental angiogenesis. A literature review highlights endoglin as a well-known mesenchymal stem cell marker responsible for vascular development and angiogenesis. NGS analysis showed that endoglin overexpression in DEHP-exposed MDA-MB-231 cells correlated with tumor development and growth. An in vivo zebrafish xenograft assay showed that VEGFA induced sprouting of the subintestinal vein (SIV) in embryos injected with DEHP-exposed cells. Endoglin knockdown reduced SIV sprouting and VEGFA expression in zebrafish embryos. An in vitro HUVEC tube formation assay showed that endoglin depletion reversed DEHP-induced VEGF-mediated HUVEC tube formation in coculture. DEHP-induced endoglin activated TGFβ/SMAD3/VEGF and MAPK/p38 signaling in MDA-MB-231 cells. A cytokine angiogenesis antibody array showed induced expression of the inflammatory cytokines IL1α, IL1β, IL6, and IL8, along with GMCSF and VEGF. Endoglin knockdown reversed DEHP-induced activation of the TGFβ/SMAD3/VEGF signaling axis, MAPK/p38 signaling, and cytokine regulation, limiting angiogenesis potential both in vivo and in vitro. Targeting endoglin might serve as a potential alternative treatment to control angiogenesis, leading to metastasis and limiting cancer progression.

## 1. Introduction

In the growth and progression of breast cancer, new blood vessel generation from pre-existing vessels is pivotal and is known as neovascularization. An increase in angiogenic cues and mutation at a genetic level are some of the factors that accompany the growing tumor. In quiescent vessels, endothelial cells (ECs) line up by forming a lumen surrounded by a thick layer of pericytes or vascular smooth muscle cells (VSMCs), supporting the structural integrity of the vessel [1]. Angiogenic cues or ischemia increase endothelial permeability, giving rise to matrix metalloproteins for extracellular matrix degradation and relieving pericyte-EC contact [2]. This provides space and a chance for the adjacent cells to influx fluids and/or macromolecules due to the absence of cell-to-cell contact. Through coordinated activation, endothelial cells tend to proliferate and migrate toward promigratory phenomena (such as VEGF) to reach their final destination, where they undergo morphogenesis to form a lumen and branches [3]. Several factors, such as VEGF, thrombin, and sphingosine 1 phosphate, control EC permeability and lead to reversible loss of junctional integrity [4]. Angiogenesis provides oxygen, nutrients, and metabolites to growing tumors simultaneously, removes waste products from them, and nourishes them to form a solid mass. Angiogenesis is mostly controlled by proangiogenic factors such as VEGF and its tyrosine kinase receptors VEGFR1 and VEGFR2. Activation of the VEGF receptor by its ligand leads to activation of a number of downstream signaling pathways, such as PI3K, MAPK, and PLCγ, which mostly occur during angiogenesis [5].

Cancer highjacks and diverts the flow of blood vessels toward it to fulfil its requirements, leading to enhanced proliferation and metastatic spread. The angiogenesis niche is triggered by hypoxia, leading to the production of angiogenic factors and immunosuppressive cytokines. For instance, in neuroblastoma (NB), extracellular microenvironment hypoxia is reported to promote extracellular adenosine generation, which induces VEGF production by binding with A3 adenosine receptor and activating HIF-1α/2α/VEGF axis [6]. In addition, a recent study conducted by Shen demonstrated that HIF-1α expression is parallel with VEGF expression, and both expressions are positively correlated with poor prognosis of ovarian cancer [7]. In contrast, pituitary adenylate cyclase-activating polypeptide (PACAP) and its receptor PAC1R suppress angiogenic pathway and mesenchymal markers, such as vimentin and MMP-2 by inhibition of MAPK/PI3K/Akt signaling in the hypoxia niche of glioblastoma [8,9]. Maugeri et al. indicated that caffeine exerts anti-tumor effects on glioblastoma multiforme (GBM), characterized by extensive hypoxia through significant downregulation of HIF-1α as well as VEGF [10]. Metformin, widely prescribed for type II diabetes, is also suggested to circumvent hypoxia-mediated overexpression of pro-angiogenic factors (VEGF) and aberrant angiogenesis through promoting perfusion [11]. In our previous study, a reduced ROS level was observed in MDA-MB-231 under prolonged exposure to DEHP [12]. Chen et al. revealed that hypoxia serves as a double-edged sword in ROS production. On one hand, HIF decreases ROS production via suppressing the tricarboxylic acid (TCA) cycle; on the other hand, it promotes ROS formation by NADPH oxidase (NOX) because NOX is a downstream gene of HIF [13]. Collectively, there is an intimate interplay between hypoxic microenvironment, ROS, and angiogenesis. Some of the angiogenic factors involved in tumor-induced angiogenesis include vascular endothelial growth factor (VEGF), platelet-derived growth factor (PDGF), fibroblast growth factor (FGF), and cytokines such as interleukins (ILs), tumor necrosis factors (TNFs), interferons (INFs), and tumor growth factors (TGFs) [14]. The process of angiogenesis is governed by activators and inhibitors [15]. Determination and localization of angiogenic activators and inhibitors can lead us to design a drug to reduce the metastatic spread rate of the respective cancer.

Endocrine disrupters (EDs)/endocrine disrupting chemicals (EDCs) are naturally occurring or synthetic compounds that mimic endocrine hormonal activity to affect the normal physiological functioning of the endocrine system [16]. EDs are often found in many products, such as plastic, cosmetics and hygiene products, personal care products, packaging, medical devices, heavy metals, and furniture [17]. One ED/EDC is phthalates or phthalic acid, which are used to enhance the durability and flexibility of plastic products and are reported to exert detrimental effects on human health [18]. Some of the studies showed its ubiquitous presence in urine in industrialized countries. Its effects range from an imbalance in the hormonal system to human sexual and reproductive system development [19]. Frequently found phthalates include diethyl phthalate (DEP), dimethyl phthalate (DMP), di (2-ethylhexyl) phthalate (DEHP), diisononyl phthalate (DINP), and dibutyl phthalate (DBP) [20]. Among all phthalates, DEHP is the most widely used; it is noncovalently bound in plastics and results in leaching under different conditions, resulting in ubiquitous environmental distribution [18,21]. Phthalates may result in human exposure in one of several ways, such as dermal contact, inhalation, and ingestion, which could pose a risk to potential health conditions, such as cancer progression and disturbance of reproductive and respiratory systems [22,23]. DEHP exposure in uterine leiomyoma cells increased cell viability and anti-apoptotic proteins [24]. In lung cancer cells, it increased cell viability, cell proliferation, and inflammatory proteins [25]. Inhibition of angiogenesis reduced placental growth and development, followed by DEHP exposure, in pregnant mice [26]. Angiogenic factors such as sFlt-1 and PIGF are affected by treatment with DEHP, suggesting a strong correlation between angiogenesis and phthalates [27]. An increase in cancer cell viability and suppression of angiogenesis adversely both affect cancer development. An increase in cancer cell viability spares a chance to proliferate and metastasize and increases the niche to develop a solid tumor. At the molecular level, several signaling pathways correlated with cancer growth and differentiation following phthalate exposure have been recorded. Zu et al. reported that phthalate exposure activated ERK5/p38 signaling, which promoted the spread of prostate cancer [28]. Activation of MAPK (ERK1/2) and JNK signaling following DBP treatment induced testicular and liver damage in a murine model [29,30]. Similarly, in our previous study, we demonstrated the involvement and activation of PI3K/Akt signaling in continuous and long-term exposure to DEHP-induced multidrug resistance in breast cancer cells [12].

Endoglin (CD105/ENG) is a transmembrane glycoprotein and a coreceptor of the tumor growth factor-β (TGFβ) family. Endoglin is highly expressed in proliferating vascular endothelial cells [31,32], and it interacts with their serine/threonine kinase receptors (TGFβRI or TGFβRII), forming a heterotrimer along with ligand [33,34,35]. Other ligands include TGFβ, activins, GDFs, and BMPs [36]. Ligand binding leads to receptor dimerization and autophosphorylation at serine residues, recruiting R-SMAD and Co-SMAD proteins to the nucleus and transcribing target genes, leading to several biological processes, namely, proliferation, migration, differentiation, cell death, and angiogenesis [37]. Significant evidence has shown increased endoglin in the TGF-β signaling pathway in tumor-associated endothelial cells, making it ‘a marker’ for tumor-induced angiogenesis [38]. Solid tumors such as prostate, cervical, and breast cancer showed an increased level of endoglin in the endothelium [39,40,41]. The expression of endoglin increases during wound healing, inflammation, vasculogenesis, and angiogenesis [42,43]. Liu et al. showed that endoglin is indispensable for VEGF-induced angiogenesis. Endoglin-deficient mouse embryos die due to malformation of blood vessels [44]. Proliferation and angiogenesis are inhibited in ovarian cancer endothelial cells following endoglin depletion/knockdown [45]. In murine mammary carcinoma, endoglin silencing reduces the growth and number of vessels formed during tumor progression [46]. Endoglin interacts with VEGFR2 in a VEGF-dependent manner to sustain and stabilize VEGFRII on the cell surface to promote tip cell formation for tumor progression and growth [47]. In the current study, we demonstrated the detailed mechanism of endoglin-mediated regulation of TGFβ, MAPK/p38 signaling, and cytokines controlling angiogenesis in prolonged DEHP-exposed MDA-MB-231 cells in vitro and in vivo.

## 2. Materials and Method

### 2.1. Cell Culture

MDA-MB-231 cells, human breast cancer cells, and human umbilical vein endothelial cells (HUVECs) were used as experimental models for the study. MDA-MB-231 cells were acquired from the American Type Culture Collection (ATCC) and maintained in DMEM (Gibco, Grand Island, NE, USA) supplemented with penicillin, streptomycin antibiotics, 10% FBS, 0.03% glutamine, and 1 mM sodium pyruvate in a 5% CO_2_ incubator at 37 °C in humidified conditions. A sample of 293T cells were acquired from ATCC and maintained in low antibiotic (0.1% penicillin/streptomycin) DMEM containing FBS (10%), glutamine (0.03%), and sodium pyruvate (1 mM). Human umbilical vein endothelial cells (HUVECs) were purchased from Lonza (CC2519, Basel, Switzerland) and maintained in EGM^TM^-2 endothelial cell growth medium (CC-3121, Lonza, Basel, Switzerland) supplemented with an EGM^TM^-2 supplement pack (CC-4122, Lonza, Basel, Switzerland) containing bovine brain extract (BBE) and free of exogenous VEGF in a 5% CO_2_ incubator at 37 °C in humidified conditions.

### 2.2. Reagents and Antibodies

DEHP (Sigma-Aldrich, 36735, Saint Louis, MO, USA) was reconstituted in DMSO (Sigma-Aldrich, Saint Louis, MO, USA). Doxorubicin (Dox) (Sigma-Aldrich, D1515, Saint Louis, MO, USA) was dissolved in DMSO (Sigma-Aldrich, Saint Louis, MO, USA). Primary antibodies for proteins: Endoglin/CD105 (71 kDa, Proteintech, 10862-1-AP, Rosemont, IL, USA), MAPK (ERK1/2) (44 kDa, Cell Signaling, 137F5, Danvers, MA, USA), phospho-MAPK (ERK1/2) (Thr202/Tyr204) (44 kDa, Cell Signaling, 9101 s, Danvers, MA, USA), p38 (38 kDa, Proteintech, 14064-1-AP, Rosemont, IL, USA), phospho-p38 (Tyr182) (38 kDa, Santa Cruz, SC-166182, Santa Cruz, CA, USA), GAPDH (35.5 kDa, EMD Millipore, MAB374, Burlington, MA, USA), Smad2/3 (52, 60 kDa, Cell Signaling, 8685, Danvers, MA, USA), phospho-Smad2 (Ser465/467)/Smad3 (Ser423/425) (52, 60 kDa, Cell Signaling, 8828, Danvers, MA, USA), β actin (43 kDa, Santa Cruz, SC-47778, Santa Cruz, CA, USA), and VEGFA (43 kDa, Proteintech, 66828-1-IG, Rosemont, IL, USA). Secondary antibodies: Goat anti-rabbit IgG (Alexa Fluor 594, Invitrogen, A11012, Waltham, MA, USA) was used as the secondary antibody.

### 2.3. DEHP Exposure and Stable Clone Establishment

MDA-MB-231 cells were exposed to DEHP at a low concentration (100 nM) for three months in cell culture medium. DEHP stock was directly diluted in culture medium to achieve a final concentration of 100 nM. Cells were cultured in 100 mm cell culture dish supplemented with 10–12 mL of DEHP containing DEHP. Upon reaching 80–90% confluence, ¼ cells were passaged and continuous culture with DEHP treatment was maintained for 3 months. After 3 months, DEHP treatment was terminated permanently, and cells were treated with 10 nM Dox for 72 h. Colonies formed following dox treatment were selected and maintained for further study. Untreated MDA-MB-231 cells are denoted as the control (Ctrl), and DEHP-exposed cells are denoted as DEHP from here onward. Both untreated and DEHP-treated cells were maintained and equally passaged (10–12 passages) to reduce senescence effects.

### 2.4. Zebrafish Xenograft

Transgenic zebrafish strain *Tg (fli1: EGFP)* was raised and maintained at 28.5 °C in the Zebrafish Core Facility at KMU. Embryos were obtained by pairwise mating and incubating them in 0.03% phenylthiourea (PTU) at 28.5 °C in an incubator for 48 h. Forty-eight hpf embryos were injected with Vybrant^®^ DiI-stained control and DEHP-exposed MDA-MB-231 cells using a microinjector setup. Following injection, embryos were maintained in an incubator at 28.5 °C for 24 h. Embryos were further observed for SIV sprouting, and images were captured under a fluorescence microscope (MZ10F, Leica, Singapore) using Metaview software (version 7.8.0.0).

### 2.5. RNA Sequencing

To evaluate the underlying mechanism of DEHP-induced angiogenesis in MDA-MB-231 cells, next-generation sequencing (NGS) was performed on the control and DEHP-exposed MDA-MB-231 cells as described in our previous publication [12]. Briefly, 3 μg of isolated RNA from Biotools Co. Ltd. (Taipei, Taiwan) was used for sequencing. RNA was sequenced, and data were analyzed by Illumina software. DEGs and GO were analyzed by TopHat (v2.0.12) and GoSeq & topGO (2.12); KEGG analysis was performed by KOBAS (v2.0). For data confirmation and validation, the log ratio of expression obtained by NGS was further evaluated by QIAGEN Ingenuity Pathway Analysis (IPA^®^, QIAGEN Redwood, Redwood City, CA, USA, Available online: www.qiagen.com/ingenuity (accessed on 21 December 2021).

### 2.6. Lentiviral Transfection

Envelop plasmid (pMD2. G), packaging plasmid (pCMV-dR8.91), and short hairpin RNA (shRNA) containing hairpin-pLKO.1 vector was used for lentiviral particle preparation; pMD2. G, pCMV-dR8.91, scrambled/mock shRNA (clone ID: ASN0000000004) and shENG (clone ID: TRCN0000083140) were purchased from the RNAi core facility (Academia Sinica, Taipei, Taiwan). Scramble shRNA or sheng, along with pMD2. G and pCMV-dR8.91, were transfected into 293T cells using Lipofectamine 2000 (Thermo Fisher, 11668019, Waltham, MA, USA) in OptiMEM for 18 h. Packaged lentiviruses were harvested in FBS/BSA-enriched DMEM at 36 h and 48 h post-transfection. Collected lentiviruses were concentrated using a 100 K molecular weight cutoff filter unit (MAP100C38, Pall Corporation, New York, NY, USA). Lentiviral transfection with shScr (mock) or shENG containing lentivirus was performed using Lipofectamine 2000 in the control and DEHP-treated MDA-MB-231 cells for 24 h. Transfected cells were exposed to puromycin (1–2 µg/mL) for selection and establishment of stable knockdown cells.

### 2.7. Quantitative Polymerase Chain Reaction (qPCR)

Total RNA was extracted from 25–30 noninjected and tumor cell-injected embryos using TRIzol reagent. Similarly, cellular RNA from the control and DEHP-exposed (mock-treated and ENG knockdown) cells was isolated using TRIzol reagent. Extracted RNA was reverse transcribed to cDNA, and qPCR was performed using SYBR Green master mix (Applied Biosystems^TM^, Waltham, MA, USA). cDNA from zebrafish embryos or cells and zebrafish-specific primers against VEGFa (XM_009292018.3) (5′-CAGTTATTTCTCGCGGCTCT-3′; 5′- TCCCCCTTCTTTGGGTATGT-3′) and GAPDH (NM_001115114.1) (5′-GTGGAGTCTACTGGTGTCTTC-3′ and 5′-GTGCAGGAGGCATTGCTTACA-3′) and human-specific primers against endoglin (NM_001278138.2) (5′-CGCCAACCACAACATGCAG-3′ and 5′- GCTCCACGAAGGATGCCAC-3′); TGFβRII (NM_003242.6) (5′-GCTTTGCTGAGGTCTATAAGGC-3′ and 5′-GGTACTCCTGTAGGTTGCCCT-3′); SMAD3 (NM_001145103.2) (5′-GTCTGCAAGATCCCACCAG-3′ and 5′-AGCCCTGGTTGACCGACT-3′); β-actin (NM_001101.5) (5′-TGAGACCTTCAACACCCCAGCCAT-3′ and 5′-CGTAGATGGGCACAGTGTGGGTG-3′) was purchased from Genomics (New Taipei, Taiwan) and analyzed on a QuantStudio^TM^ 5 Real-Time PCR System (A28574, Applied Biosystems^TM^, Waltham, MA, USA). The data analysis was performed using QuantStudio^TM^ 5 Design & Analysis Software (version 1.5.1, Applied Biosystems^TM^, Waltham, MA, USA), and relative mRNA expression was calculated by the equation 2^−ΔΔCt^. Data quantification was performed by SigmaPlot (version 12.3, Systat Software, Inc., Erkrath, Germany).

### 2.8. Western Blotting

Protein samples (30–40 µg) were separated by SDS–PAGE, followed by transfer to PVDF membranes. PVDF membrane blocking was performed in 5% non-fat milk and subsequent incubation with specific primary antibodies against ENG, MAPK (ERK1/2), phospho-MAPK (ERK1/2), phospho-p38, and GAPDH at 4 °C. The following day, membranes were washed with blocking buffer to remove unbound antibodies, and host-specific secondary antibody treatment was performed. Finally, membranes were washed, and chemiluminescence signal detection was performed using an ECL^TM^ detection kit and luminescent image analyzer (Amersham Imager 680, GE Healthcare and Bioscience ab, Princeton, NJ, USA).

### 2.9. In Vitro-Angiogenesis/Tube Formation Assay

In vitro angiogenesis/tube formation was performed by coculturing HUVECs with the control and DEHP-treated MDA-MB-231 cells. Briefly, 1 × 10^4^ HUVECs were seeded on Matrigel (Culturex^®^ 5X BME)-coated 24-well plates. A total of 1 × 10^5^ of the control and DEHP-exposed cells were seeded in 0.4 µm Transwell inserts (3413, Corning Incorporated, Corning, NY, USA) and placed in HUVEC seeded plates. Cultures were incubated at 37 °C in an incubator, and images were captured 8 h after seeding. Tube formation data analysis was performed by web-based WIMASIS Image Analysis software (Onimagin Technologies SCA, Cordoba, Spain). Data quantification was performed by SigmaPlot.

### 2.10. Quantitative Enzyme-Linked Immunosorbent Assay (ELISA)

Approximately 2 × 10^5^ of the control and DEHP-exposed (mock-treated and ENG knockdown) MDA-MB-231 cells were seeded in 6-well plates and incubated overnight. After incubation, serum-free culture medium was replaced, and cells were incubated for 48–72 h. Culture medium was collected and used to perform VEGF quantification using a Human VEFG_165_ Standard TBM ELISA Development kit (900-T10, PEPROTech, Hamburg, Germany), as described previously [48].

### 2.11. Immunofluorescence

Approximately 2 × 10^4^ of the control and DEHP-exposed (mock-treated and ENG knockdown) MDA-MB-231 cells were seeded in cell culture chamber slides (30114, SPL Lifesciences, Pocheon-si, Korea) and incubated overnight at 37 °C in an incubator. Cells were fixed with 4% paraformaldehyde (PFA), permeabilized with 0.1% Triton X-100, and blocked with blocking buffer (3% BSA in PBS). Cells were incubated with primary antibody (SMAD3), washed with blocking buffer, and incubated with host-specific secondary antibody (goat anti-rabbit IgG, Alexa Fluor 594), along with nuclear staining with DAPI. Fluorescence imaging was performed on a fluorescence microspore (Olympus, 1X71, Shinjuku, Japan). Data analysis and quantification were performed by SigmaPlot (version 12.3, Systat Software, Inc., Erkrath, Germany).

### 2.12. Antibody Angiogenesis Array

An antibody angiogenesis microarray was performed using a Human Angiogenesis Antibody Array-membrane kit (ab193655, Abcam, MA, USA), according to the manufacturer’s instructions. Briefly, control and DEHP-exposed (mock-treated and ENG knockdown) MDA-MB-231 cells were lysed, and the protein concentration was estimated; 200–250 µg of total protein was used. The protein-conjugated membranes provided in the kit were blocked with blocking buffer for 30 min. After incubation, half of the blocking buffer was removed and replaced with an equal volume of protein sample and then incubated for 1.5–5 h at room temperature. Membranes were washed and conjugated with a biotinylated antibody cocktail for 1.5–2 h at room temperature. Membranes were washed and incubated with HRP-conjugated streptavidin for 2 h at room temperature. Finally, membranes were incubated with detection buffer, and chemiluminescence signals were detected by a luminescence imager analyzer (Amersham Imager 680). Quantification of signal intensity was performed by ImageJ (version 1.53, NIH, USA). Data quantification was performed using SigmaPlot.

### 2.13. Statistical Analysis

Statistical analysis of data was performed by SigmaPlot. Standard one-way ANOVA and Student’s t tests were used to compare different groups. Data presented is as a ±standard deviation (SD). A *p* value was considered statistically significant if it was <0.05.

## 3. Results

### 3.1. Prolonged DEHP Exposure Enhances the Angiogenesis Potential of MDA-MB-231 Cells In Vivo

**Figure 1:**
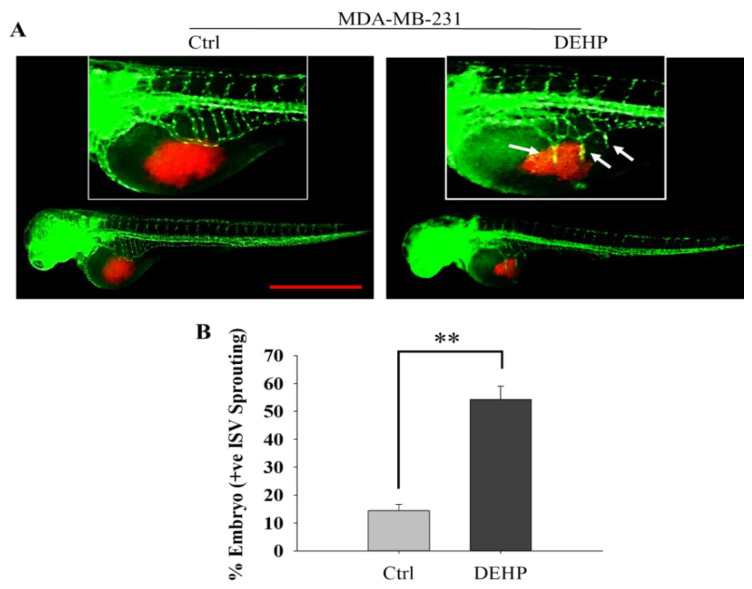
Representative and quantitative results of the zebrafish xenograft angiogenesis assay. (**A**) DEHP-exposed MDA-MB-231 cells enhance SIV sprouting in *Tg (fli1: EGFP)* zebrafish embryos at 24 hpi. Red fluorescence: DiI stained breast cancer cells; Green fluorescence: vascular network of zebrafish embryo. Scale bar = 1000 µm. White arrow: Indicate site of SIV sprouting. (**B**) Quantitative results of angiogenesis in zebrafish showing SIV sprouting (*n* = 50); ** *p* < 0.001.

To evaluate the effect of prolonged DEHP exposure at physiological concentrations, a zebrafish xenograft assay was performed. Control and DEHP-treated MDA-MB-231 cells injected in 48 hpf Tg (fli1: EGFP) embryo sacs showed enhanced SIV sprouting in DEHP-treated MDA-MB-231-injected embryos compared to control MDA-MB-231-injected embryos (Figure 1A). On average, 55% and 12% of embryos (*n* = 50) showed SIV sprouting in DEHP-treated and control MDA-MB-231-injected embryos, respectively (Figure 1B). A 43% difference in SIV sprouting is indicative of pro-angiogenic effects of DEHP exposure in MDA-MB-231 cells.

### 3.2. Endoglin Predicted as a Regulator of DEHP-Induced Angiogenesis in Breast Cancer Cells

**Figure 2:**
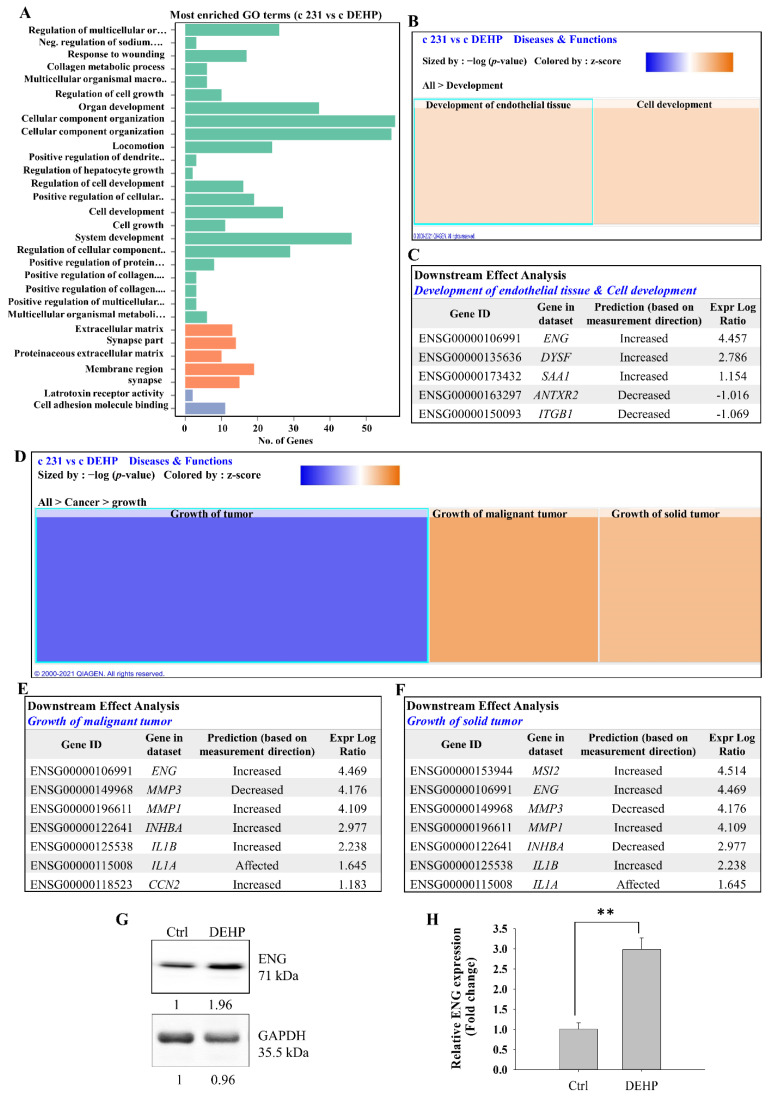
Representative results of RNA sequencing and IPA of control and DEHP-treated MDA-MB-231 cells. (**A**) Bar chart of significantly enriched GO terms and the number of DEGs enriched in biological processes (green), cellular components (orange), and molecular function (violet). (**B**) IPA-derived heatmap analysis of development under cellular diseases and functions of the DEGs involved. (**C**) Downstream analysis of genes involved in the development of endothelial tissue and cell development. (**D**) IPA-derived heatmap analysis of cancer growth under cellular diseases and functions of the DEGs involved. (**E**,**F**) Downstream analysis of genes involved in the growth of malignant tumors and the growth of solid tumors in control and DEHP-exposed MDA-MB-231 cells, highlighting the involvement of endoglin/CD105 and its prediction as a regulator of DEHP-induced angiogenesis. (**G**,**H**) Prolonged DEHP exposure increased endoglin expression at the protein and mRNA level in MDA-MB-231 cells; ** *p* < 0.001.

To identify the underlying mechanism and regulators of DEHP-induced angiogenesis potential in MDA-MB-231 cells, RNA sequencing was performed. Gene ontology (GO) enrichment analysis of differentially expressed genes (DEGs) was performed for control and DEHP treated MDA-MB-231 cells. GO enrichment analysis showed the 30 most significantly enriched terms related to biological process, cellular component, and molecular function. Regulation of cell growth, organ development, regulation of cell development, system development, locomotion, response to wounding, and cell growth were identified among the top 30 enriched GO terms (Figure 2A). GO terms such as cellular growth, organ development, system development, and locomotion are closely related to angiogenesis or vascular development, which is the initial phase of system or organ development. During cancer progression, angiogenesis is a prerequisite for tumor growth and metastasis. We further used IPA, a bioinformatics tool to identify and predict the profile of gene expression and pathways underlying the impact of prolonged DEHP exposure to breast cancer cells, and IPA analysis showed the significant enrichment of endothelial tissue development and cell development under disease and function (Figure 2B). Downstream effect analysis highlighted the involvement of endoglin with a log expression ratio of 4.457 (Figure 2C). Further disease and function analysis found enrichment of growth of malignant tumors and growth of solid tumors (Figure 2D). Interestingly, endoglin was found to be positively correlated with both the growth of malignant tumors and the growth of solid tumors. To our surprise, the matrix metalloproteins MMP1 and MMP3, along with the inflammatory cytokines IL1β and IL1α, were predicted to be activated with a high log expression ratio (Figure 2E,F). The expression of endoglin was further confirmed through Western blotting and qPCR. Prolonged DEHP exposure increased the expression of endoglin almost 2-fold at protein and 3-fold at mRNA levels (Figure 2G,H). Collectively, endoglin was predicted to be involved in endothelial tissue and cell development, along with malignant and solid tumor growth, indicating a central role of endoglin. Considering the pathophysiological conditions, angiogenesis/vascular development plays a crucial role in normal tissue development or tumor growth [49,50]. An increase in endoglin expression consistent with prediction of NGS data points towards its involvement in regulating DEHP-induced angiogenesis, tumor growth, and ultimately metastasis.

### 3.3. Endoglin Depletion Reverses the DEHP-Induced Angiogenic Potential of MDA-MB-231 Cells

**Figure 3:**
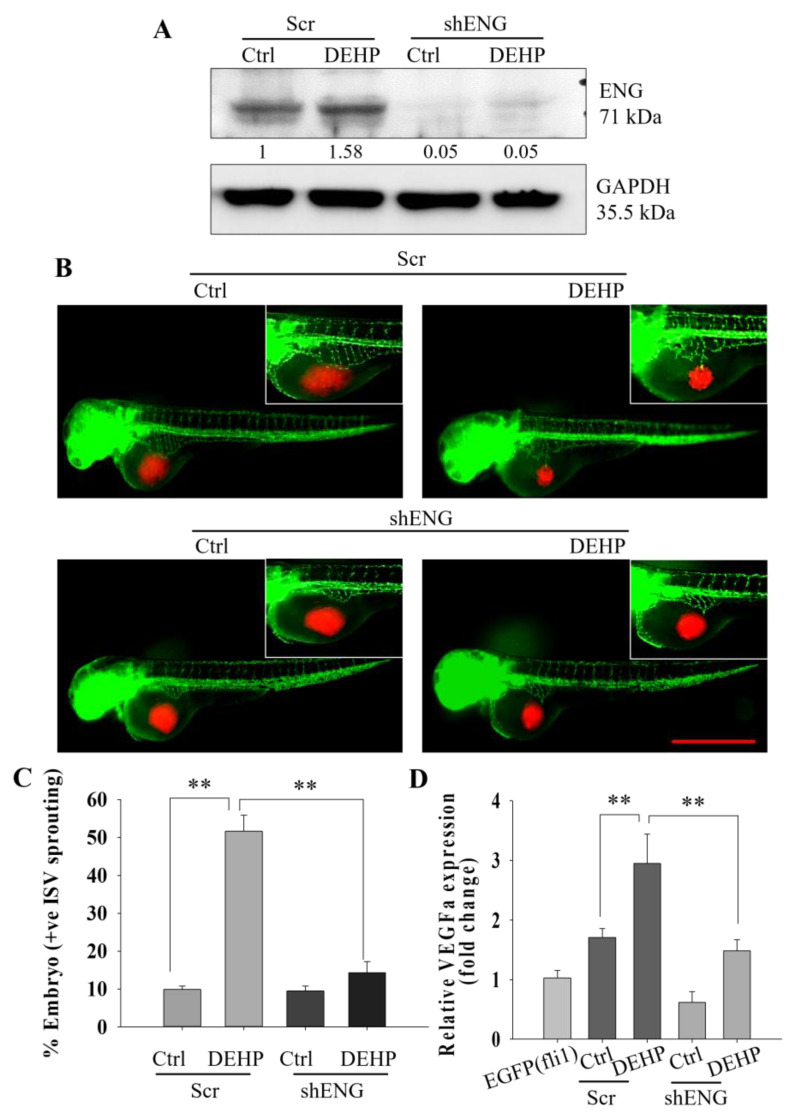
Representative and quantitative results showing that DEHP-induced endoglin-controlled VEGFA-mediated angiogenesis in vivo. (**A**) Prolonged DEHP treatment upregulated endoglin expression in MDA-MB-231 cells, and shENG treatment depleted endoglin expression in control and DEHP-exposed MDA-MB-231 cells. (**B**) Endoglin knockdown reduced DEHP-induced SIV sprouting in Tg (fli1: EGFP) zebrafish embryos at 24 hpi. Scale bar =1000 µm. (**C**) Quantitative results of angiogenesis showing a reduced number of zebrafish embryos showing SIV sprouting following endoglin knockdown (*n* = 50). (**D**) Depletion of VEGFA mRNA levels in zebrafish embryos following endoglin knockdown cell xenografts of DEHP-exposed MDA-MB-231 cells; ** *p* < 0.001.

To confirm and validate the involvement of endoglin in DEHP-induced angiogenesis in MDA-MB-231 cells, endoglin was knocked down using lentivirus-mediated shRNA transfection. Western blotting showed upregulation of endoglin in scrambled/mock shRNA-treated DEHP-exposed MDA-MB-231 cells. However, endoglin expression was completely depleted in shENG-treated control and DEHP-exposed MDA-MB-231 cells (Figure 3A). Next, control and DEHP-exposed MDA-MB-231 cells (mock- and shENG-treated) were injected into the embryo sacs of 48 hpf Tg (fli1: EGFP) embryos. SIV sprouting evaluation at 24 h after injection showed that endoglin knockdown significantly reduced SIV sprouting (Figure 3B). The number of embryos showing SIV sprouting following xenografting was reduced from 50% to 12.5% in endoglin knockdown DEHP-exposed MDA-MB-231 cell-injected mock-treated embryos compared with DEHP-exposed MDA-MB-231 cell-injected embryos (Figure 3C). However, no change was observed in mock- and shENG-treated control MDA-MB-231 cells. To understand the mechanism of cancer cell-induced SIV sprouting of Tg (fli1: EGFP) embryos, mRNA levels of zebrafish VEGFA were investigated. qPCR results showed that VEGFA mRNA levels were significantly increased in breast cancer cell-injected embryos compared to embryos without injection. The highest VEGFA mRNA levels were recorded in mock-treated DEHP-exposed MDA-MB-231 cell-injected embryos, followed by embryos injected with mock-treated control MDA-MB-231 cells. Interestingly, DEHP knockdown significantly reduced VEGFA mRNA levels in embryos injected with shENG-treated DEHP-exposed MDA-MB-231 cells (Figure 3D). Surprisingly, ENG knockdown in control cell-injected embryos showed no significant change in VEGFA mRNA levels. Overall, endoglin controls and regulates DEHP-induced SIV sprouting and VEGFA expression in Tg (fli1: EGFP) embryos injected with breast cancer cells.

### 3.4. Endoglin Regulates HUVEC Tube Formation through VEGF Production in Prolonged DEHP-Treated MDA-MB-231 Cells

**Figure 4:**
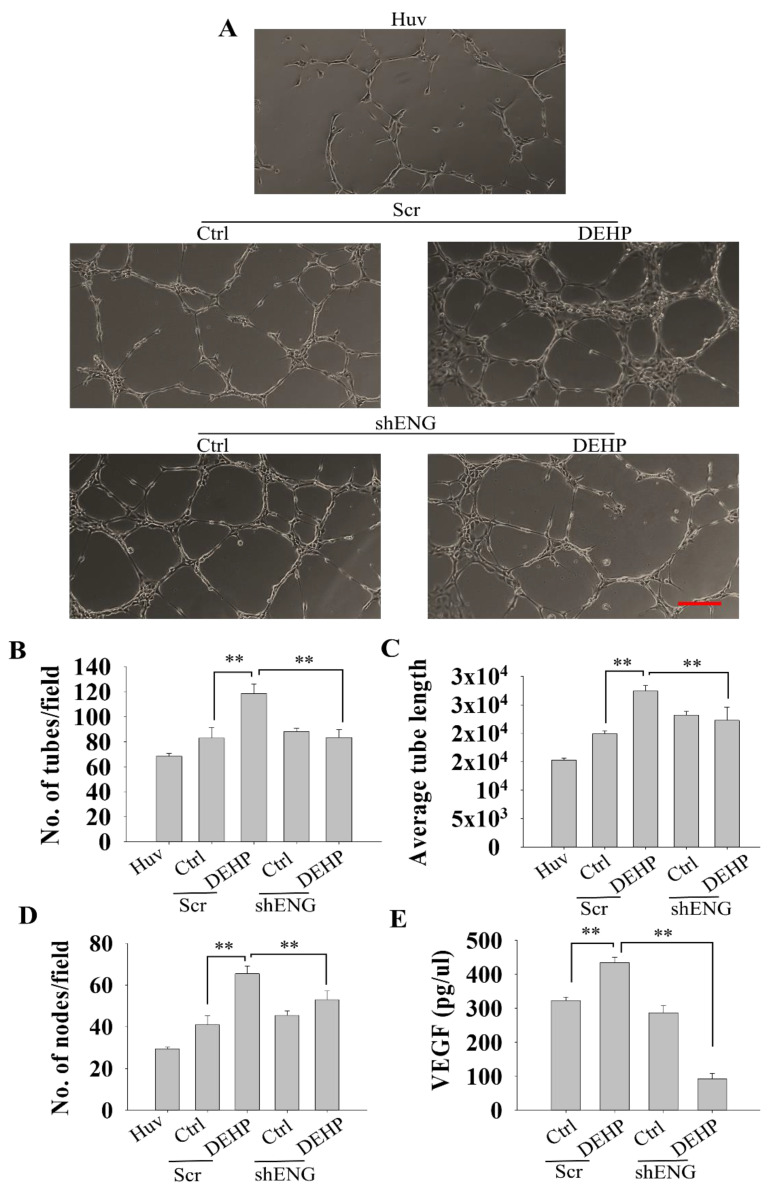
Representative and quantitative results of endoglin-mediated HUVEC tube formation. (**A**) Induced HUVEC tube formation in coculture with control and DEHP-exposed MDA-MB-231 cells at 8 h after seeding; endoglin knockdown reversed DEHP-induced HUVEC tube formation. Scale bar = 250 µm. (**B**) Quantitative evaluation of the number of tubes formed in coculture at 8 h after cell seeding. (**C**) Quantitative evaluation of average tube length in coculture at 8 h after cell seeding. (**D**) Quantitative evaluation of the number of nodes formed in coculture at 8 h after cell seeding. (**E**) Results of quantitative ELISA for VEGF levels in the cell culture medium of control and DEHP-exposed MDA-MB-231 cells (mock- and shENG-treated); ** *p* < 0.001.

To validate the results of the in vivo zebrafish angiogenesis assay, we performed an in vitro angiogenesis assay by coculturing HUVECs with control and DEHP-exposed MDA-MB-231 cells (mock- and shENG-treated). Induced tube formation was observed under all coculture conditions compared to HUVECs alone, and mock-treated DEHP-exposed MDA-MB-231 cell coculture showed the highest tube formation potential (Figure 4A). Endoglin knockdown in DEHP-exposed cells significantly reduced HUVEC tube formation, consistent with the in vivo results. The number of tubes, average tube length, and number of nodes showed similar observations. The average number of tubes, average tube length, and number of nodes formed were highest in mock-treated DEHP-exposed MDA-MB-231 cells, and HUVECs without coculture were lowest among all groups. Endoglin knockdown in DEHP-exposed MDA-MB-231 cells significantly reduced the average number of tubes, average tube length, and number of nodes formed in coculture. Interestingly, endoglin knockdown in control MDA-MB-231 cells showed a slight increase in the average number of tubes and average tube length; however, it did not affect the number of nodes formed in coculture (Figure 4B–D). To understand the mechanism of HUVEC tube formation in coculture, quantitative ELISA was performed. Cell culture medium of control and DEHP-exposed MDA-MB-231 cells (mock- and shENG-treated) was used to evaluate VEGF concentrations. The results showed that mock-treated DEHP-exposed MDA-MB-231 cell culture medium contained the highest VEGF concentration of 432 pg/µL, followed by 320 pg/µL in mock-treated control MDA-MB-231 cell culture medium. Endoglin knockdown in DEHP-exposed MDA-MB-231 cells resulted in significant depletion of VEGF levels, as low as 97 pg/µL. However, endoglin knockdown did not affect VEGF levels in control MDA-MB-231 cells (Figure 4E). Collectively, prolonged DEHP exposure induced HUVEC tube formation in coculture through endoglin-mediated VEGF production.

### 3.5. Endoglin Maintains the TGFβ/SMAD3/VEGF Signaling Axis in Prolonged DEHP-Treated MDA-MB-231 Cells

**Figure 5:**
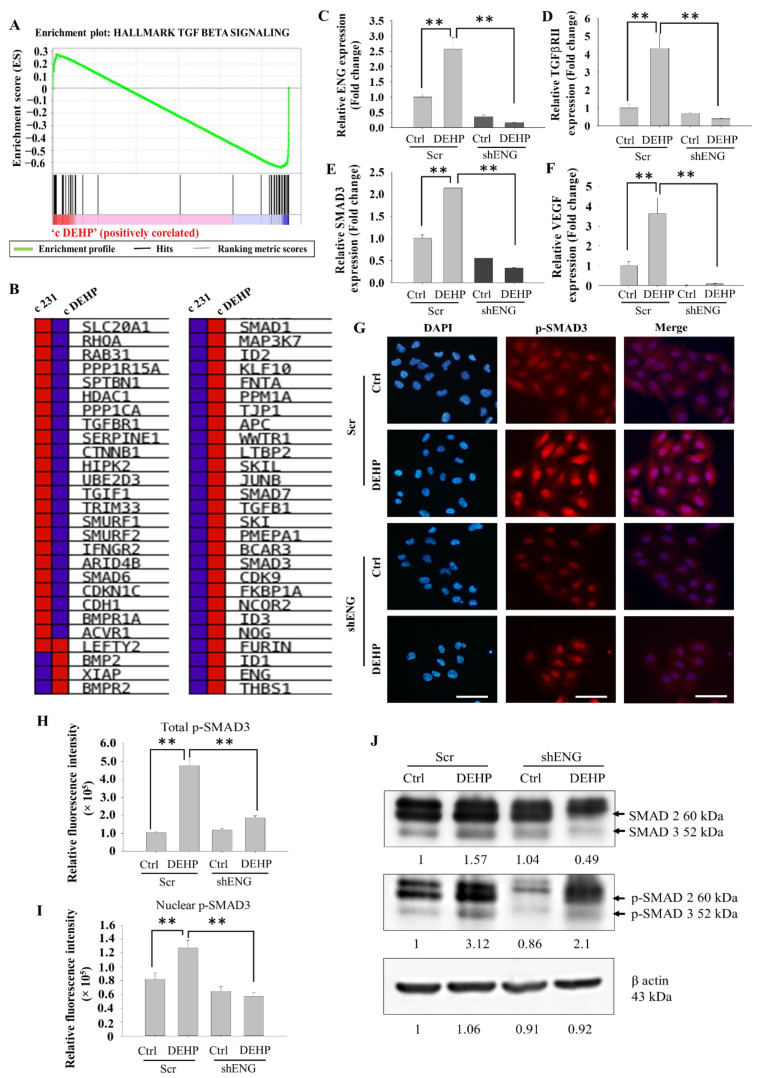
Representative and quantitative results of endoglin-mediated regulation of the TGFβ/SMAD3/VEGF signaling axis. (**A**) GSEA of TGFβ signaling showing a positive correlation with DEHP-treated MDA-MB-231 cells (enrichment score). (**B**) Blue–Pink O’ gram showing core enrichment of individual genes in the TGFβ signaling gene set (red: upregulation; blue: downregulation). (**C**–**F**) qPCR analysis showing the mRNA expression of endoglin, TGFβRII, SMAD3, and VEGF in control and DEHP-exposed MDA-MB-231 cells (mock- and shENG-treated). (**G**) IF results showing expression changes and nuclear localization of p-SMAD3. Scale bar = 100 µm. (**H**,**I**) Quantitative analysis of p-SMAD3 IF showing total and nuclear p-SMAD3 expression levels. (**J**) SMAD3 and phospho-SMAD3 expression evaluated by Western blotting; ** *p* < 0.001.

To understand the underlying mechanism of DEHP-induced angiogenesis potential through VEGF expression, gene set enrichment analysis (GSEA) was performed. TGFβ signaling was enriched and found to be positively correlated with DEHP-exposed MDA-MB-231 cells, with an enrichment score of 0.28 compared to −0.67 of the control MDA-MB-231 cells (Figure 5A). Individual DEGs of the TGFβ gene set showed core enrichment in DEHP-treated MDA-MB-231 cells (Figure 5B). Endoglin is a TGFβ coreceptor, and endoglin upregulation following DEHP treatment is postulated to stabilize and induce TGFβ signaling, supporting the results. mRNA levels of TGFβ signaling markers TGFβRII and SMAD3, along with endoglin and VEGF, were evaluated and showed that DEHP treatment increased mRNA levels of endoglin, TGFβRII, SMAD3, and VEGF; however, endoglin knockdown resulted in a significant decline in mRNA levels of endoglin, TGFβRII, SMAD3, and VEGF (Figure 5C–F). qPCR results show that activation of canonical TGFβ/SMAD3/VEGF signaling regulated through endoglin in DEHP-exposed MDA-MB-231 cells, consistent with GSEA results. To verify the transcriptional regulatory activity of SMAD3, IF was performed, and consistent with prior observations, SMAD3 expression was highest in DEHP-exposed MDA-MB-231 cells. SMAD3 was observed to be colocalized in the nucleus of DEHP-exposed cells, indicating SMAD3 activation and nuclear translocation. Interestingly, endoglin knockdown not only reduced the expression of SMAD3 but also depleted nuclear translocation, limiting its activation and SMAD3-mediated transcriptional regulation of VEGF (Figure 5G–I). The effect of DEHP exposure and endoglin knockdown was evaluated on the expression of total and phospho-SMAD3 and showed that DEHP exposure upregulates total and phospho-SMAD3. Consistent with qPCR and IF findings, endoglin knockdown downregulates expression of both total and phospho-SMAD3 in DEHP-exposed MDA-MB-231 cells (Figure 5J). Overall, prolonged DEHP exposure induces endoglin expression, which stabilizes TGFβRII and activates TGFβ signaling, resulting in increased VEGF production and secretion and enhancement of the angiogenesis potential of MDA-MB-231 cells.

### 3.6. Endoglin-Mediated MAPK/p38 Signaling and Secretory Cytokine Production May Contribute to DEHP-Induced Angiogenesis in MDA-MB-231 Cells

**Figure 6:**
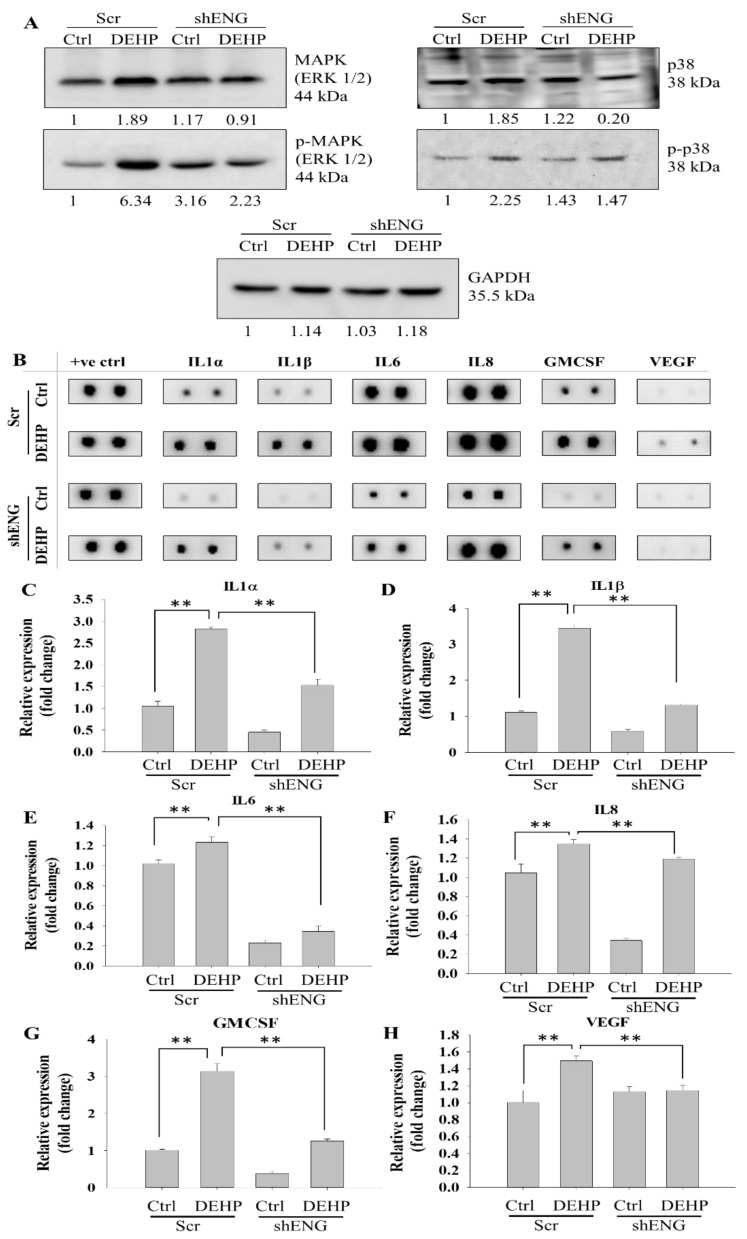
Representative and quantitative results of endoglin-mediated MAPK/p38 signaling and cytokine regulation. (**A**) Western blotting results showing upregulation of MAPK, phospho-MAPK, p38, and phospho-p38 in DEHP-treated MDA-MB-231 cells, and endoglin reversed protein expression. (**B**) Representative results of the antibody angiogenesis array showing endoglin-mediated expression of the inflammatory cytokines GMCSF and VEGF. (**C**–**H**) Quantification of the expression of IL1α, IL1β, IL6, IL8, GMCSF, and VEGF in control and DEHP-exposed MDA-MB-231 cells (mock- and shENG-treated). +ve ctrl: positive control; ** *p* < 0.001.

Western blotting was performed to evaluate the effect of prolonged DEHP treatment on MDA-MB-231 cells and showed activation of MAPK/p38 signaling. Upregulation of MAPK, phospho-MAPK, and phospho-p38 was observed in DEHP-treated cells compared to control MDA-MB cells. Endoglin knockdown reduced the expression of MAPK, phospho-MAPK, and phospho-p38 in DEHP-exposed MDA-MB-231 cells; however, endoglin knockdown in control MDA-MB-231 cells upregulated all three proteins (Figure 6A). Overall, these results indicate that endoglin can positively regulate MAPK/p38 signaling in DEHP-exposed cells and that MAPK/p38 may not be involved in angiogenesis progression in control MDA-MB-231 cells. Next, to understand the molecular mechanism of MAPK/p38 signaling, we performed an angiogenesis antibody array to screen multiple candidate proteins that might be involved in ENG-mediated angiogenesis in MDA-MB-231 cells. The results showed six proteins involved in endoglin-mediated angiogenesis among the panel of 43 angiogenesis-associated markers evaluated. The inflammatory cytokines IL1α, IL1β, IL6, IL8, GMCSF, and VEGF were highly expressed in DEHP-treated cells compared to control MDA-MB-231 cells. Endoglin knockdown reversed the expression of all six markers in both control and DEHP-exposed MDA-MB-231 cells (Figure 6B–H). Overall, it can be predicted that endoglin-mediated MAPK/p38 signaling may regulate the expression of the abovementioned inflammatory cytokines and that GMCSF contributes to DEHP-induced angiogenesis potential in MDA-MB-231 cells. Moreover, in control MDA-MB-231 cells, endoglin knockdown resulted in MAPK/p38 activation, but it reduced the expression of cytokines that might not be interrelated with each other. Endoglin depletion still affected the expression of secretory cytokines in control MDA-MB-231 cells, which may be regulated by different signaling mechanisms, warranting further investigation.

## 4. Discussion

Di-2-ethylhexyl phthalate (DEHP) is widely used as a plasticizer in plastic products and is ubiquitous in daily life [51]. In particular, Chinese populations are exposed to higher phthalate concentrations than Western populations, leading to exposure from contaminated food and air [52]. A large amount of evidence suggests that DEHP exposure is intimately correlated with several disorders, such as Alzheimer’s disease, male infertility, and increased risk of breast cancer [53,54,55]. The effects of DEHP exposure in vivo has been evaluated in several studies. Barkat et al. demonstrated that high oral DEHP doses in pregnant mice resulted in infertile male offspring [56]. However, other groups found prenatal DEHP exposure affected female reproduction in F1–F3 generations, leading to reproductive aging [57]. These studies indicate that DEHP exposure exerts significant effects on reproductive system in vivo; however, long-term effects with low concentrations of DEHP have not been demonstrated. A strategic study of cancer progression in long-term DEHP-exposed mice might provide an insight into physiological condition and the effects of DEHP exposure on cancer progression. Moreover, this type of study might lack the actual prolonged DEHP exposure to tumor cells, considering the proliferation rate of tumor implants in vivo. To overcome this issue, we exposed breast cancer cells with physiological DEHP concentration (100 nM) long term in an attempt to mimic real life conditions. In addition, phthalate has been confirmed to promote cancer progression through induced proliferation, drug resistance, and angiogenesis. Crobeddu et al. demonstrated that DEHP and its primary metabolite mono (2-ethylhexyl) phthalate (MEHP) enhance the proliferation of human breast ductal carcinoma cells via upregulation of progesterone receptor (PR) [55].

In our previous study, we showed that long-term DEHP exposure at a concentration can induce multidrug resistance through increased expression of ABC transporters and reduced intracellular ROS generation [12]. Over the past few decades, most studies have reported the role of DEHP in placental development [26,58]. However, the association between DEHP and angiogenesis in cancer remains largely unknown. Tsai et al. reported that benzyl butyl phthalate (BBP) can induce angiogenesis in hepatocellular carcinoma (HCC) through the aryl hydrocarbon receptor AhR/ERK/VEGF axis [59]. This finding indicates that phthalate has the potential to promote angiogenesis. Consistent with previous reports, our data showed that SIV sprouting was remarkably increased in zebrafish embryos implanted with prolonged DEHP-exposed MDA-MB-231 cells. We demonstrated the proangiogenic activity of DEHP treatment to mimic physiological DEHP exposure for an extended period of time because most DEHP-related studies emphasize high-concentration DEHP treatment for a short period and research on low-dose and prolonged DEHP exposure–mediated tumor progression is relatively insufficient.

Endoglin, also known as CD105, is a transmembrane glycoprotein that serves as a coreceptor for TGF-β receptors I and II and is important for angiogenesis [60]. Kasprzak et al. revealed that endoglin plays a significant role in angiogenesis in HCC. It cannot only promote the proliferation of liver sinusoidal endothelial cells (ECs) but also enhance the resistance of ECs to apoptosis [61]. Endoglin is also reported to promote colorectal cancer liver metastasis by cancer-associated fibroblast (CAF)-expressing endoglin and TGF-β signaling [62]. Consistent with these findings, our study showed that endoglin knockout reduced SIV sprouting and VEGFa expression in zebrafish embryos. Moreover, endoglin depletion seriously disrupted DEHP-induced VEGF-mediated HUVEC tube formation. Collectively, DEHP-induced angiogenesis is mainly mediated by endoglin.

A literature review revealed that endoglin is the auxiliary receptor of the TGF-β receptor that facilitates the initiation of the TGF-β/SMAD signaling pathway. Phosphorylated SMAD serves as a transcription factor (TF) to promote the expression of the downstream effector genes responsible for cell proliferation, migration, and angiogenesis [63]. SMAD3 functions as a TF once phosphorylated following TGFβ signaling activation, which induces the expression of its downstream transcripts, especially VEGFA [64,65]. Our results indicated that prolonged DEHP exposure activated the TGFβ/SMAD3/VEGF signaling axis. Moreover, DEHP treatment induced nuclear localization of SMAD3, indicating the transcriptional regulator activity of SMAD3. Endoglin depletion reversed the activation of TGFβ signaling and reduced the nuclear translocation of SMAD3.

Mitogen-activated protein kinase (MAPK) is an important signaling pathway involved in several cellular events, such as cell differentiation, proliferation, inflammation and apoptosis. There are three major components of the MAPK cascade, including c-Jun N-terminal kinases (JNKs), p38, and extracellular signal-regulated kinases (ERKs) [66,67]. Preliminary evidence indicates that VEGFA overexpression is triggered by both the PI3K and MAPK/p38 signaling pathways, which further drives angiogenesis [68]. Our results corroborated the activation of MAPK/p38 in DEHP-exposed MDA-MB-231 cells. Conversely, endoglin knockdown inhibited phosphorylation and activation. Taken together, DEHP exposure can induce angiogenesis through ENG-controlled TGF-β/SMAD3/VEGF and MAPK/p38 signaling.

Accumulating studies have suggested that MAPK/p38 is also involved in the production of cytokines, including IL-1 and IL-6, and granulocyte-macrophage colony-stimulating factor (GM-CSF) [69,70,71]. In proteome profiling, the relationship between inflammation and angiogenesis was apparently observed; in addition, IL-1β and VEGF had considerable overlaps on biological functions and signal transduction of pro-angiogenic effects, especially with both of them activating MAPK in HUVECs [72]. Another finding suggested that IL-1β also plays a crucial role in invasiveness and metastasis. Angiogenesis is capable of providing sufficient nutrients because metastasis requires abundant energy to complete [73]. Interleukin-6 (IL-6), a pleiotropic cytokine, has been suggested to induce angiogenesis in mounting studies. IL-6 can contribute to not only the upregulation of VEGF but also increased endothelial tip cell sprouting through the pSTAT3 and jagged-1/Notch-3 signaling pathways, respectively [74]. Gopinathan et al. highlighted that IL-6 shares similarities with VEGF on pro-angiogenic function; however, the difference lies in the fact that a vessel with aberrant pericytes coverage was observed in IL-6-induced angiogenesis, but was absent in the VEGF-stimulated one [75]. Our results indicated that prolonged DEHP exposure induced the expression of the inflammatory cytokines IL1α, IL1β, IL6, IL8, and GM-CSF through endoglin regulation in MDA-MB-231 cells.

## 5. Conclusions

**Figure 7:**
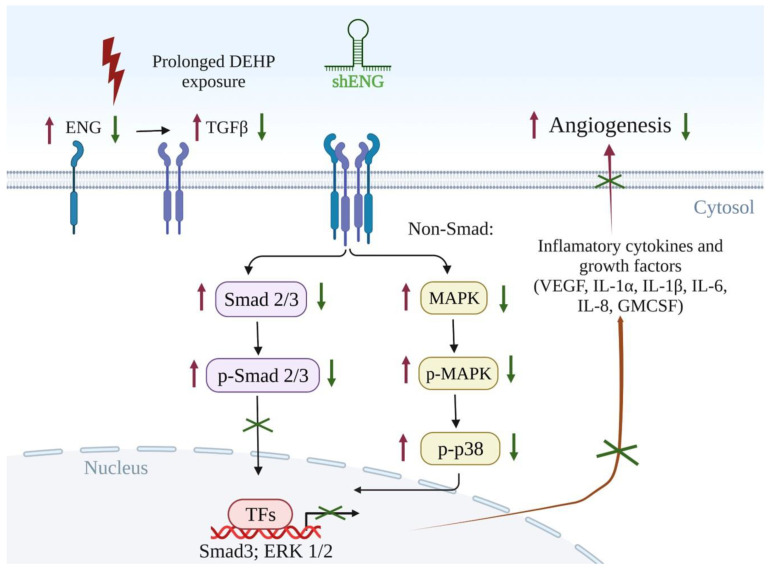
Schematic representation of prolonged DEHP exposure-induced angiogenesis potential in breast cancer cells. Prolonged DEHP exposure at physiological concentrations upregulate the expression of endoglin (ENG). Endoglin overexpression activates TGFβ and MAPK/p38 signaling-mediated production of VEGF; inflammatory cytokines IL1α, IL1β, IL6, IL8; and GMCSF, contributing to enhanced DEHP-induced angiogenesis potential in breast cancer cells. shRNA mediated ENG knockdown reversed ENG induced angiogenesis through downregulation of TGFβ and MAPK/p38 signaling along with reduced expression of inflammatory cytokines IL1α, IL1β, IL6, IL8; and GMCSF.

In conclusion, our study confirmed that prolonged DEHP exposure can promote angiogenesis in TNBC via the ENG/TGFβ/SMAD3/VEGF signaling axis in vitro and in vivo. Alternatively, long-term DEHP exposure can also induce upregulation of the inflammatory cytokines IL1α, IL1β, IL6, and GM-CSF through the MAPK/p38 signaling pathway to facilitate breast cancer progression. Hence, targeting ENG and its downstream signaling pathway might be a promising strategy to normalize vasculature or curb angiogenesis and subsequently inhibit the progression of breast cancer (Figure 7).

## Data Availability

The authors confirm that the data supporting the findings of this study are available within the article and will be provided on request.
